# Osteopetrosis; a report of two Iranian patients with autosomal recessive inheritance pattern

**Published:** 2012

**Authors:** Saeid Morovvati, Sara Amirpour Amraii, Hosna Zahed Shekar Abi, Nastaran Shahbazi, Reza Ranjbar

**Affiliations:** 1 *Research Center for Human Genetics, Baqiyatallah University of Medical Sciences, Tehran, Iran.*; 2 *Tehran Medical Unit, Islamic Azad University, Tehran, Iran.*; 3 *Molecular Biology Research Center, Baqiyatallah University of Medical Sciences, Tehran, Iran.*

**Keywords:** Osteopetrosis, autosomal recessive, consanguinity

## Abstract

In the rare hereditary bone disorder of osteopetrosis, reduced bone resorption function leads to both the development of densely sclerotic fragile bones and progressive obliteration of the marrow spaces and cranial foramina. Marrow obliteration, typically associated with extramedullary hemopoiesis and hepatosplenomegaly, results in anemia and thrombocytopenia; and nerve entrapment accounts for progressive blindness and hearing loss. Severe infantile or malignant osteopetrosis is the worst type of the disease which has poor prognosis. In this study we report two cases of severe infantile or malignant type of the disease in an Iranian family.

Our two patients were children of a family where the wife is a grandchild of the husband’s aunt. The first patient had episodes of seizure and spastic in extremities 2 weeks after birth. Gradually, the patient showed upper and lower respiratory problems and horizontal nystagmus. X-Ray of hand and foot showed widening and increased bone density and physical examination showed hepatosplenomegallay and petechiae in extremities. The patient expired due to cardiopulmonary arrest. The second patient had also episodes of seizure 2 weeks after birth. Gradually, dissymmetry in eyes appeared and blindness was confirmed by ophthalmologist. Finally the patient expired because of severe pneumonia.

Autosomal recessive osteopetrosis has been reported in most ethnic groups although it is more frequently seen in ethnic groups where consanguinity is common. We report for the first time two cases of severe infantile or malignant type of the disease in an Iranian family.

Osteopetrosis, a rare hereditary bone disorder is also named marble bone disease because of the dense rock-like appearance of the bone in the disease (1). Several genes have been associated with osteopetrosis in humans (2). It is thought that bone formation is normal and that bone resorption is reduced, resulting in the presence of excessive calcified tissue. The abnormal resorption results in the normal structural pattern of the bone being grossly altered, the cortices are thickened, individual bony trabeculae are increased, and the marrow spaces are encroached upon leading to a paucity of haemopoietic tissue with consequent secondary anemia (3). Four types of osteopetrosis have been described: Severe infantile or malignant type, osteopetrosis with renal tubular acidosis and cerebral calcifications, benign type and intermediate type (2). Severe infantile or malignant type is the worst one and has poor prognosis. In this study we report two cases of severe infantile or malignant type of the disease in an Iranian family.

## Case presentation

**Fig 1:**
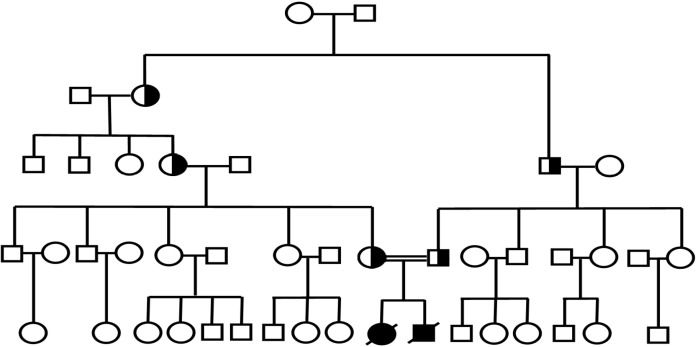
Pedigree of patients

We are reporting two patients which are offsprings of an Iranian family where the wife is a grandchild of the husband’s aunt (Fig.1).

**Patient one**

He was born with normal vaginal delivery. Weight at birth was 3000 grs. Two weeks after birth, the infant had episodes of seizure like eye staring, focal movement in face and spastic in extremities. Infant underwent work-up and hypocalcaemia was detected (Ca=3.5). Gradually, the patient showed upper and lower respiratory problems and when he was 2 years old he had tonsillectomy due to tonsillar hypertrophy. At this time, patient didn’t gain catch-up weight and his weight was approximately 9.2 Kg when he was 2.5 years old. The patient had horizontal nystagmus, but CT scan and serial head circumference didn’t reveal abnormality. X-Ray of hand and foot showed widening and increased bone density, and loss of the normal corticomedullary differentiation. Then, patient showed productive rough, high fever and confusion and was hospitalized showing hepatosplenomegallay upon examination and petechiae in extremities (Platelet: 61000). Patient was admitted in ICU and intubated, but finally cardiopulmonary arrest occurred and he expired.

**Patient two**

The second patient was born with normal vaginal delivery. Her height, weight and head circumference was 46cm, 3700grs and 35cm respectively. She had normal apgar score and infantile reflexes were normal. At the fifth day after birth she became icteric. Some blood indexes of the patient were Hct:43, WBC:9100, Plts:145000. In the course of hospitalization, she had two episode of seizure. In clinical testes hypocalcaemia (Ca=5) was detected. Gradually, the mother of the patient discovered dissymmetry in eyes of patient, and the patient gol hospitalized. The ophthalmologist confirmed blindness, but the two retinas were normal. In the course of disease she had not good weight (weight at 4 years old was 1Kg) and she was edentulous. Then, an ulcerative lesion was found in right mandible of the patient that gradually increased in size and the patient became supportive. Patient became febrile and hospitalized with diagnosis of chronic osteomyelitis.In the course of hospitalization the patient presented cough, respiratory distress and the level of consciousness decreased, so she was intubated but finally expired because of severe pneumonia.

## Discussion

The growth and remodeling of bone that occurs during vertebrate development requires the carefully balanced activities of bone-forming osteoblast cells and bone-resorbing osteoclast cells. Disruption of this dynamic equilibrium can lead to a variety of pathological states. A century ago, Albers-Schönberg described the radiographic findings of osteopetrosis (4) including a generalized increased bone density together with modelling defects at the metaphyseal ends of long bones, resulting in typically widened and blunted diaphyses and metaphyses (5). Autosomal recessive malignant osteopetrosis (ARO) is a rare congenital disorder of bone resorption. It is caused by the failure of osteoclasts to resorb immature bone. Defective resorption leads to both the development of densely sclerotic fragile bones (osteosclerosis) and progressive obliteration of the marrow spaces and cranial foramina. Marrow obliteration, typically associated with extramedullary hemopoiesis and hepatosplenomegaly, results in anemia and thrombocytopenia; and nerve entrapment accounts for progressive blindness and hearing loss.Other major manifestations are failure to thrive, pathological fractures, and increased infection rate (6). 

The generation of superoxide by peripheral blood leucocytes is defective in patients with osteopetrosis. This, along with the anemia, poor nutrition, recurrent hospital admissions, and the frequent ear, nose, and throat complications, results in a greatly increased susceptibility to infections especially pneumonia and septicemia, which are a common cause of death (7). Infantile malignant osteopetrosis becomes apparent during the first months of life. The natural course of ARO is characterized by early mortality: only 30% of children are still alive at the age of 6 years, the mortality rate being higher in the first 2 years of life. Without treatment, life expectancy rarely exceeds twenty years. The main causes of death are severe bone marrow failure and overwhelming infections (8-9).

The incidence of ARO is approximately 1 in 300,000 births but is almost 10 times as high in Costa Rica (10). Osteopetrosis has been reported in most ethnic groups although as the disease is very rare it is more frequently seen in ethnic groups where consanguinity is common and consan-guineous sibships with multiple affected patients have been described in Costa Rica [9], Kuwait (11) and Saudi Arabia (12). 

Osteopetrosis is a genetically heterogeneous disease. Several human genes have been described as the cause of ARO. The T-cell immune-regulator-1 (TCIRG1) gene (MIM 604592), which is mutated in about 50 to 60 percent of the patients, results in defects in the A3 subunit of the osteoclast vacuolar H^+^-ATPase proton pump (13-14). This gene has been mapped to 11q13 (15). The chloride channel 7 (CLCN7) gene (MIM 602727) which accounts for about 10 to 15 percent of cases, encodes an osteoclast-specific chloride channel. Heterozygous CLCN7 mutations cause a wide range of phenotypes even in the same family, ranging from early severe to nearly asymptomatic forms (16). Recessive osteopetrosis with renal tubular acidosis (MIM 259730) which accounts for a small proportion of patients with osteopetrosis, results from a mutation in the gene encoding carbonic anhydrase type II (CAII) and a defect in production of carbonic acid and proton (17).

Several patients have been reported with the grey-lethal gene (OSTM1) mutation, coding for a cytoplasmic protein involved in OCL functional activity, but this mutation also occurs in few children with osteopetrosis (18). Mouse GL protein function is absolutely required for osteoclast and melanocyte maturation and function. Perturbation of this balance can lead to a reduction of bone mass, as seen in osteoporosis, or to an abnormal accumulation of bone, as in osteopetrosis. It should be noted that a substantial percentage of patients with osteopetrosis have no identifiable gene defect. The analysis of the OSTM1 gene in two patients, both from Kuwait, showed homozygosity for two nucleotide deletion in exon 2, leading to a frameshift and premature termination. The third (Lebanese) patient showed a single point mutation in exon 1, leading to a nonsense mutation (19).

Gene analysis in two Portuguese families affected with osteopetrosis showed homozygosity for CLCN7 mutations. Direct sequencing of the CLCN7 gene in both patients revealed homozygosity for two mutations G203D and P470Q (20). All nine Costa Rican patients had either one or both of the two missense mutations G405R and R444L (21).
